# Ficolin-2 binds to serotype 35B pneumococcus as it does to serotypes 11A and 31, and these serotypes cause more infections in older adults than in children

**DOI:** 10.1371/journal.pone.0209657

**Published:** 2018-12-26

**Authors:** K. Aaron Geno, Brady L. Spencer, Sejong Bae, Moon H. Nahm

**Affiliations:** 1 Division of Pulmonary, Allergy, and Critical Care Medicine, Department of Medicine, University of Alabama at Birmingham, Birmingham, Alabama, United States of America; 2 Division of Preventive Medicine, Department of Medicine, University of Alabama at Birmingham, Birmingham, Alabama, United States of America; 3 Department of Microbiology, University of Alabama at Birmingham, Birmingham, Alabama, United States of America; Public Health England, UNITED KINGDOM

## Abstract

Among 98 serotypes of *Streptococcus pneumoniae*, only a small subset regularly causes invasive pneumococcal diseases (IPD). We previously demonstrated that serotype 11A binds to ficolin-2 and has low invasiveness in children. Epidemiologic data suggested, however, that serotype 11A IPD afflicts older adults, possibly indicating reduced ficolin-2-mediated immune protection. Therefore, we studied the epidemiology of ficolin-2-bound serotypes. We obtained IPD case data from the United States Centers for Disease Control and Prevention. We studied three prominent ficolin-2-bound serotypes and their acetyltransferase-deficient variants for ficolin-2 binding and ficolin-2-mediated complement deposition with flow-cytometry. We determined the age distributions of these serotypes from the obtained epidemiologic data. We discovered that the serotype 35B capsule is a novel ficolin-2 ligand due to O-acetylation via WciG. Ficolin-2-mediated complement deposition was observed on serotypes 11A and 35B but not serotype 31 or any O-acetyl transferase deficient derivatives of these serotypes. Serotypes 11A, 35B, and 31 cause more IPD among older adults than children. Studies of the three serotypes provide additional evidence for ficolin-2 providing innate immunity against IPD. The skewed age distribution of the three serotypes suggests that older adults have reduced ficolin-2-mediated immunity and are more susceptible to these serotypes.

## Introduction

*Streptococcus pneumoniae* (the pneumococcus) is a commonly-carried commensal that normally resides in the nasopharynx. Occasionally, however, it can invade the host and cause severe infections such as sepsis and meningitis, which are together referred to as invasive pneumococcal diseases (IPD). IPD occur disproportionately among young children and older adults. As IPD are almost always caused by pneumococci expressing polysaccharide capsule [1], the capsule is recognized as the most important pneumococcal virulence factor. Extensive studies have characterized 98 distinct capsule types (serotypes) so far [2, 3], but serotypes differ in their invasiveness. Ten to thirteen invasive serotypes are responsible for most of the invasive pneumococcal diseases (IPD) among children, and pneumococcal conjugate vaccines (PCVs) have been designed to provide protection against those serotypes [4, 5]. Clinical use of PCVs has reduced IPD by the vaccine serotypes in children and, through herd immunity, in older adults [6, 7]. In contrast to invasive serotypes, some serotypes have low invasiveness; that is, they are carried in the nasopharynx often but infrequently cause IPD. For example, serotype 11A is common in carriage among children but accounts for little IPD in this population [8].

A likely explanation for the low invasiveness of serotype 11A among children is innate immunity. We and others demonstrated that the serotype 11A capsular polysaccharide is a ligand for ficolin-2 [8, 9]. Also known as L-ficolin, ficolin-2 is an innate serum protein that binds to acetyl groups of many different molecules [10] and initiates the lectin pathway of complement activation [11, 12]. Indeed, ficolin-2 can activate complement on serotype 11A bacteria and opsonize them for phagocytes [8]. We also showed that ficolin-2 binds to O-acetylation on the serotype 11A capsule, which is added to the capsular repeat unit by the O-acetyl transferase WcjE, but does not bind to serotype 11E [8], whose capsular genes are genetically identical to those of serotype 11A except for the inactivation of *wcjE* [13]. Furthermore, genetic studies of serotype 11E isolates showed *wcjE* inactivation events to be unique to each isolate [14]. These findings, taken together, suggest that serotype 11A microevolves into serotype 11E in the blood during IPD in each individual through inactivation of *wcjE* and subsequent loss of O-acetylation to escape immune pressure by ficolin-2 [14, 15]

These observations can explain the low invasiveness of serotype 11A in children [6, 13]. However, serotype 11A IPD occurs among older adults in the United States [6]. This epidemiologic observation suggests that ficolin-2 mediated immune protection is insufficient in older adults. To evaluate this hypothesis, we first investigated serotype 35B for ficolin-2 binding since serotype 35B has an O-acetyltransferase deficient counterpart named serotype 35D [3] that exhibits a similar pattern of O-acetyltransferase gene inactivation as serotype 11E [3, 16, 17]. We then determined the most prevalent ficolin-2-binding serotypes and assayed their respective abilities to activate complement. Finally, we then examined the age distributions of IPD by the serotypes that bind to ficolin-2 [8].

## Methods

The use of serum obtained from a consented healthy volunteer was approved by the University of Alabama at Birmingham Intitutional Review Board for Human Use, protocol X150323001.

### Bacterial strains

Pneumococcal isolates used in this study are described in Table 1. Serotype 35B isolate 3009–06, serotype 35D isolate 3431–06, and their isogenic derivatives expressing various *wciG* alleles are described elsewhere [3], as are serotype 11A isolate JC03 and its isogenic *wcjE* deletion derivative (i.e., serotype 11E) JC04 [18]. Serotype 31 isolate BLS133 was derived from the serotype 31 clinical isolate MNK0189 by transformation with a mutant *rpsL* allele conferring streptomycin resistance. BLS138 is an *wcjE* deletion mutant of BLS133 (i.e., expressing experimental serotype 31X1) and was created by allelic exchange mutagenesis using Janus cassette [19]. All other strains were obtained from the Statens Serum Institut, Copenhagen, Denmark. Strains were cultivated on commercial blood agar plates (ThermoFisher R01202) at 37°C with 5% CO_2_ or in Todd-Hewitt broth with 0.5% yeast extract (THY) at 37°C for liquid cultivation. Working stocks were prepared by inoculating THY from fresh overnight plates and growing to mid-log phase (OD_600_~0.6 in a 1 cm cuvette), mixing with an equal volume of fresh medium, supplementing with sterile glycerol to a final concentration of 16%, and freezing in aliquots at -80°C.

**Table 1:** Pneumococcal strains used in this study.

Strain	Serotype	Relevant Traits	Reference
3009–06	35B	Clinical serotype 35B isolate	[3]
KAG1014	35D	3009–06Δ*wciG*	[3]
3431–06	35D	Clinical serotype 35D isolate	[3]
KAG1019	35D	KAG1014 expressing *wciG* of 3431–06	[3]
KAG1022	35B	3431–06 expressing serotype 35C *wciG*	[3]
KAG1023	35B	KAG1014 expressing serotype 35C *wciG*	[3]
JC03	11A	Serotype 11A parent	[18]
JC04	11E	JC03Δ*wcjE*	[18]
MNK0189	31	Serotype 31 parent	This Study
BLS131	31	MNK0189 with mutant *rpsL* allele	This Study
BLS138	31X1^1^	BLS131Δ*wcjE*	This Study

^1^”X” designates this an experimental serotype. This serotype has not been found in nature.

### Ficolin-2 binding studies

Ficolin-2 binding was assayed by flow cytometry after incubation in normal human serum obtained from a volunteer who provided written consent (University of Alabama at Birmingham, Institutional Review Board for Human Use Protocol X150323001). Assays were performed as previously described [8] with the exception that the ficolin-2 binding reaction was carried out in gelatin veronal buffer (GVB; 142 mM NaCl, 0.15 mM CaCl_2_, 0.5 mM MgCl_2_, 0.1% gelatin, 5 mM sodium barbital, 0.004% NaN_3_, pH = 7.4). Where indicated, C-polysaccharide (Cell Wall Polysaccharide “multi”, Statens Serum Institut, Copenhagen, Denmark) or serotype 35B polysaccharide (Statens Serum Institut) was added in the indicated concentrations.

### Complement studies

Bacteria were prepared as for ficolin-2 binding studies above and incubated with 50% supernatant from a previously-described ficolin-2-expressing cell line [20, 21] at 4°C for 1 hour with agitation, washed by centrifugation, suspended in 10% C1q-depleted serum (Complement Technologies, Tyler, TX) in GVB, and incubated at 37°C with agitation for 1 hour. Cells were washed, stained with a FITC-conjugated anti-C4b/c (ThermoFisher PA128407, 1:100 dilution), and analyzed by flow cytometry.

### Statistical analysis of epidemiologic data

Data for IPD instances per serotype were obtained through a request to the United States Centers for Disease Control and Prevention (CDC). Population at risk was calculated from the CDC Active Bacterial Core surveillance coverage areas and case rates for age groups as stated in annual surveillance reports (https://www.cdc.gov/abcs/reports-findings/survreports/spneu98.html and https://www.cdc.gov/abcs/reports-findings/survreports/spneu99.html, accessed February 21, 2018). Frequencies were analyzed for statistical significance using Fisher’s exact test analyses in SAS. Data from 1998–1999 were studied to avoid confounding factors due to the impact of conjugate vaccines.

## Results

### Ficolin-2 binds to serotype 35B in a WciG-dependent manner

**Fig 1:**
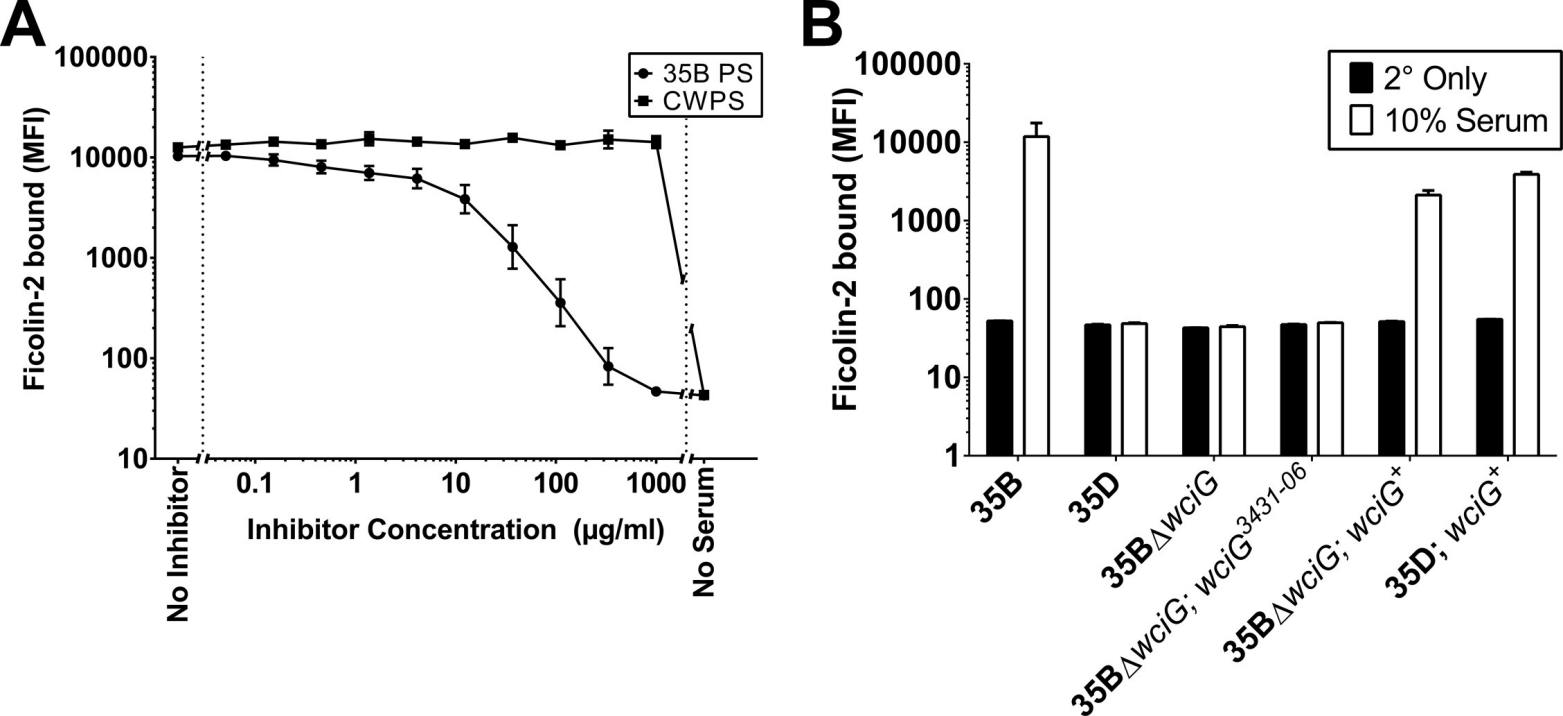
Ficolin-2 binds the serotype 35B capsular polysaccharide in a manner dependent on WciG-mediated O-acetylation. A, Serotype 35B strain 3009–06 was incubated with 10% normal human serum in the presence of increasing amounts of purified serotype 35B capsular polysaccharide (“35B PS”) or C-polysaccharide (“CWPS”) for 1 hour at 4°C. After washing, bacteria were incubated with a ficolin-2-specific monoclonal antibody, washed, and incubated with a phycoerythrin-conjugated secondary antibody prior to washing and analysis via flow cytometry. B, Serotype 35B strain 3009–06 (“35B”) and serotype 35D strain 3431–06 (“35D”), along with their derivatives KAG1014 (“35BΔ*wciG*”); KAG1019 (“35BΔ*wciG; wciG*^*3431-06*^*”*); KAG1023 (“35BΔ*wciG*; *wciG*^*+*^); and KAG1022 (“35D; *wciG*^*+*^); were incubated with 10% serum at 4°C for 1 hour prior to detection of ficolin-2 as above (“10% Serum”). In half of wells, the anti-ficolin-2 antibody was omitted (“2° Only”). Data are presented as mean ± standard deviation for two technical replicates and representative of at least three independent experiments.

**Fig 2:**
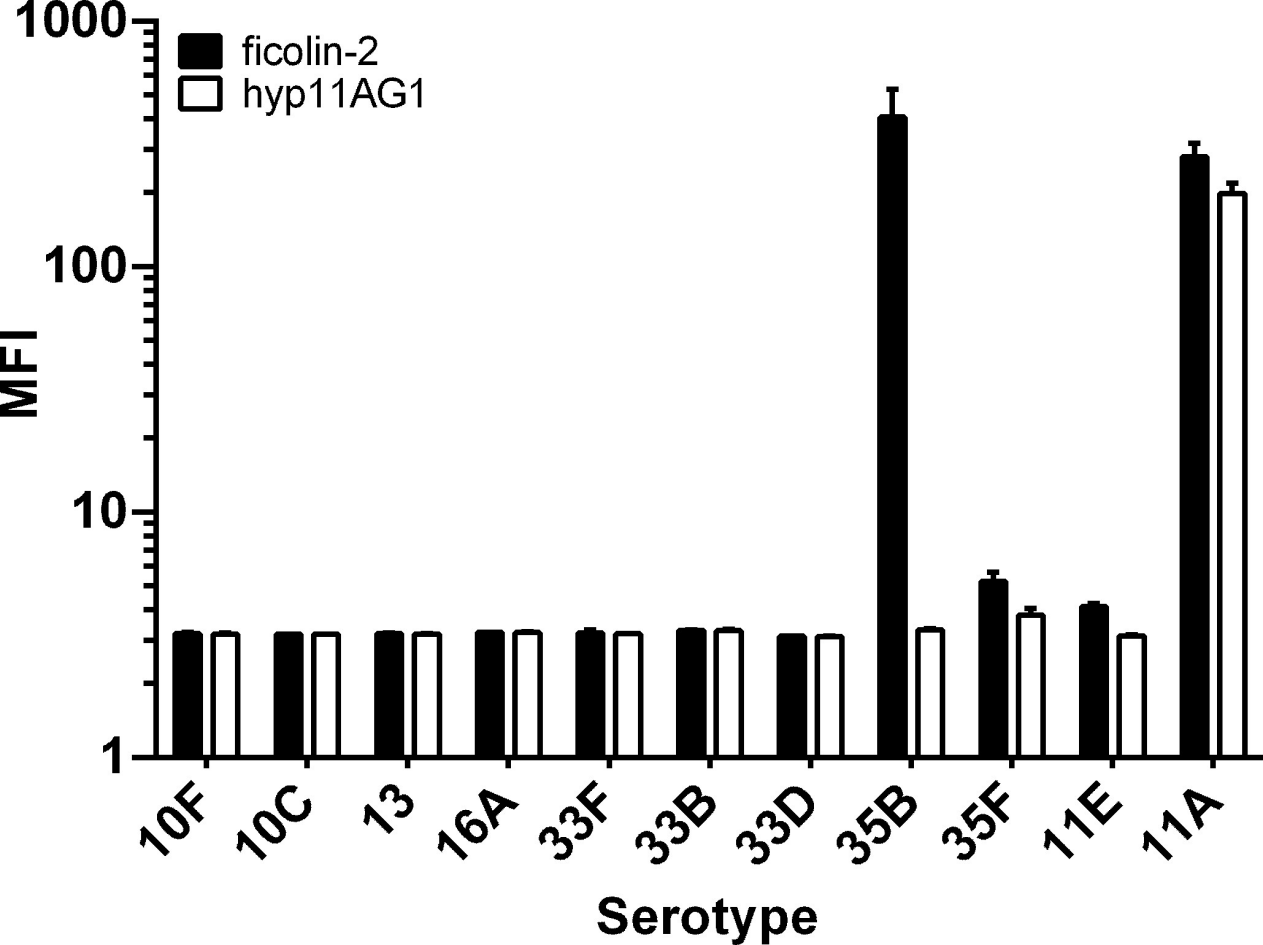
Ficolin-2 does not bind to other *wciG*-encoding serotypes. Strains of the indicated serotype were incubated with 10% normal human serum for 1 hour and stained with either an anti-ficolin-2 antibody (ficolin-2) or an isotype matched monoclonal antibody specific for the serotype 11A capsule (hyp11AG1) prior to analysis by FACS. Serotypes 11A and 11E served as positive and negative controls, respectively, for both antibodies. Data are presented as mean ± standard deviation of two technical replicates.

Serotype 35D is a recently characterized variant of serotype 35B that arises due to heterogeneous inactivation of the O-acetyl transferase gene *wciG* [3, 16, 17]. The heterogeneous *wciG* inactivations are reminiscent of the heterogeneous *wcjE* inactivations occurring during microevolution of 11A into 11E [14]. In light of these strikingly parallel findings, we examined whether ficolin-2 binds to serotype 35B. As shown in Fig 1A, ficolin-2 indeed bound to a serotype 35B clinical isolate. Binding was specific to the serotype 35B capsule, and by titrating the amount of serotype 35B polysaccharide, complete inhibition of ficolin-2 binding could be achieved. This binding was not inhibited by C-polysaccharide, a frequent contaminant of capsular polysaccharide preparations. Neither our naturally occurring serotype 35D isolate (strain 3431–06, [3]) nor a synthetic serotype 35D isolate (i.e., a serotype 35B strain in which *wciG* has been inactivated through deletion, strain KAG1014) bound to ficolin-2 (Fig 1B). Derivatives of either strain expressing a functional copy of *wciG* from outside the *cps* locus exhibited restored ficolin-2 binding, while expressing the mutant *wciG* of 3431–06 in the *wciG* deletion strain did not (Fig 1B). Ficolin-2 bound to each of the additional 24 serotype 35B isolates that we examined, including a reference isolate from Statens Serum Institut, Copenhagen, Denmark (S1 Fig). Thus, the serotype 35B capsule is a novel ficolin-2 ligand. In light of the *wciG-*dependent ficolin-2 binding of serotype 35B, we assayed reference strains for other serotypes whose *cps* loci include *wciG*, but not *wcjE*, but no ficolin-2 binding was observed apart from serotype 35B (Fig 2).

### Prevalence of ficolin-2-binding serotypes in invasive pneumococcal diseases

We obtained epidemiologic data from the United States Centers for Disease Control and Prevention (CDC) for all 14 ficolin-2-binding serotypes over the years 1998 and 1999 (i.e., prior to the introduction of PCV7), and three serotypes [11A (n = 120), 35B (n = 41), and 31 (n = 37)] were sufficiently prevalent over this period for statistical usefulness. Serotypes 20A and 20B together accounted for 34 cases of IPD over this period, but were not differentiated at that time [22, 23]; since serotype 20A binds to ficolin-2 while serotype 20B does not, we did not study these serotypes [8]. Other ficolin-2-binding serotypes were rare: three cases of IPD were caused by serotypes 15F and 33A each, and no cases were caused by serotypes 11F, 11D, 35A, 35C, 42, 43, 47F, or 47A over this period.

### Ficolin-2 supports complement deposition in vitro on serotype 35B but not serotype 35D or 31

**Fig 3:**
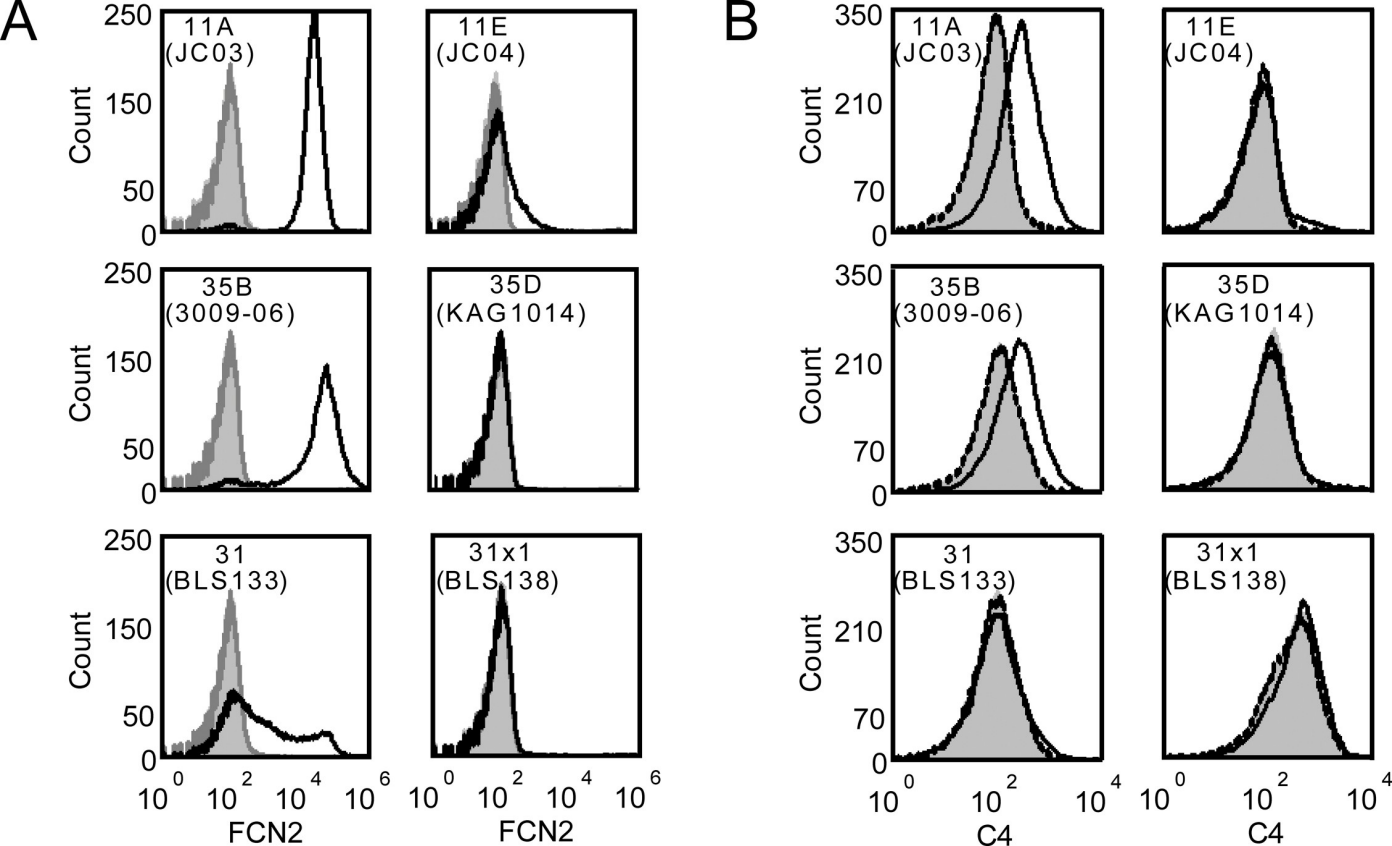
Studies of ficolin-2 binding and ficolin-2-mediated complement deposition. A, ficolin-2 (FCN2) binds to representative serotype 11A, 35B, and 31 strains but not their isogenic O-acetyl transferase-deficient derivatives (11E, 35D, and 31X1). Bacteria were incubated with buffer (light gray fill), 10% heat-inactivated normal human serum (dark gray line), or 10% normal human serum (black line) for 1 hour at 4°C. After incubation, cells were stained with a ficolin-2-specific antibody and analyzed by flow cytometry. B, ficolin-2 stimulates complement deposition on serotypes 11A and 35B but not serotype 31. Bacteria were pre-incubated with buffer (light gray shading), recombinant ficolin-2 (solid black line), or heat-inactivated recombinant ficolin-2 (dashed black line). After washing, bacteria were incubated with 10% C1q-depleted serum (C1q-dpl). Complement fragment C4 degradation products (C4b and C4c) were detected with antibodies and assayed by flow cytometry. Ficolin-2 alone (not shown) did not elicit complement deposition.

We previously demonstrated that ficolin-2 was capable of supporting complement deposition on serotype 11A bacteria in the absence of C1q (thus eliminating the classical pathway) [8], but its ability to stimulate complement opsonization on serotypes 35B and 31 were unknown. We preopsonized serotype 35B and serotype 31 bacteria, along with their isogenic O-acetyl transferase deletion strains, which do not bind ficolin-2 (Figs 1A and 3A), with recombinant ficolin-2 and incubated them in 10% C1q-depleted human serum. Serotype 35B bacteria exhibited C4b/c deposition at levels similar to the serotype 11A controls, and as expected, serotypes 11E, 35D, and 31Δ*wcjE* did not exhibit significant complement deposition (Fig 3B). However, serotype 31 exhibited no enhancement of complement deposition after preopsonization with ficolin-2. It is known that the lectin pathway activation is more challenging than the classical pathway, as different ficolin-2 molecular complexes must bring MASP1 and MASP2 in close proximity to trans-activate each other [24, 25]. Given that ficolin-2 binds to serotype 31 bacteria to a lesser degree than to serotype 11A or 35B (Fig 3B, reference [8]), this suggests that ficolin-2 may not bind to serotype 31 in sufficient density for effective complement activation in vitro where diluted serum was used as complement source.

### Serotypes 11A, 31, and 35B disproportionately afflict older adults but not children

Serotype 11A is rarely invasive in children [8], but serotype 11A IPD is more frequent in older adults [6]. To explain this, we hypothesized that ficolin-2-mediated immunity is not as effective in older adults as in children. An implication of this hypothesis is that older adults should be more susceptible than children to other ficolin-2-opsonized serotypes. We analyzed cases of IPD by ficolin-2-binding serotypes across age groups using the CDC surveillance data for the years 1998–1999 (as noted above, only serotypes 11A, 31, and 35B were sufficiently prevalent for analysis). We selected 1998 and 1999 to avoid confounding by PCV-induced alterations in serotype prevalence. Epidemiologic data for all serotypes shows the well-established high prevalence for children (i.e., individuals less than 5 years old) and older adults (i.e., individuals 65 years of age or older)(Table 2). However, the three ficolin-2 binding serotypes exhibited significantly different distributions with respect to age (*P* < 0.01) and were all but absent from infection in children under five years old (Table 2). Thus, these epidemiologic data suggest a breakdown of ficolin-2-mediated protection with age.

**Table 2:** Frequencies of invasive pneumococcal disease by serotype.

	Age (Years)						
Serotype	< 5^a^	5–34	35–49	50–64	≥ 65	Total	p-value^b^
11A	3	12	31	21	53	120	****^c^
35B	5	1	8	7	20	41	*
31	0	4	8	6	19	37	****
All other serotypes	1975	851	1429	1055	2095	7405	
Total	1983	868	1476	1089	2187	7603	
Relative prevalence (IPD per 100,000)^d^	80.1	5.7	16.2	21.4	53.0	20.4	
Population at Risk^e^ (Millions)	2.475	15.277	9.053	5.058	4.102	35.965	

^a^Data are reported as cases over the period 1998–1999 in the US.

^b^Fisher’s exact test comparing cases of indicated serotype against cases of “other serotypes” by age group.

^c^Results were statistically significant at *, *P* < 0.05; ****, *P* < 0.0001.

^d^Calculated by dividing the number of IPD in the Total row by the Population at Risk.

^e^Sum of the 1998 and 1999 surveillance areas reported by the US CDC at https://www.cdc.gov/abcs/reports-findings/survreports/spneu98.html and https://www.cdc.gov/abcs/reports-findings/survreports/spneu99.html, accessed February 21, 2018.

## Discussion

It is well known that IPD are prevalent in the extremes of age (i.e., young children and older adults). However, IPD by serotype 11A are common in older adults but relatively uncommon in children [6, 8] even though children frequently carry serotype 11A [8]. Ficolin-2 can bind the serotype 11A capsule and opsonize serotype 11A pneumococci for phagocytes. Thus, to explain the unusual epidemiologic observation with serotype 11A, we hypothesized that ficolin-2 provides protection against IPD in children but not in older adults. To strengthen this hypothesis, we examined the US epidemiology of IPD of other ficolin-2 binding serotypes in addition to serotype 11A. We found serotypes 31 and 35B caused enough IPD for meaningful analysis, and their epidemiology is similar to that of serotype 11A: their IPD are preferentially associated with older adults. It is notable that serotype 11A is included in the 23-valent pneumococcal polysaccharide vaccine, the primary vaccine given to older adults until recent years; it is unknown whether these IPD occurred in vaccinated hosts. The US epidemiologic data provide additional support for the idea that ficolin-2 provides innate protection in children but not in older adults. In addition to the US data, surveillance data from the United Kingdom obtained before the introduction of conjugate vaccines show that serotypes 11A and 31 caused more IPDs in older adults (>65 years) than in young children (<5 years) (S1 Table) [26]. Thus, while additional epidemiologic data are desirable, the altered distribution of ficolin-2-opsonized serotypes may be noticeable in other countries.

Our study also demonstrates that ficolin-2 binds to and fixes complement upon serotype 35B by targeting the acetyl group created by an O-acetyl transferase, WciG. This was unexpected because all other ficolin-2 targets on pneumococcal capsules have been so far created by another O-acetyl transferase WcjE [8]. Several pneumococcal capsule types have *wciG* in their capsular genetic loci, but, in contrast to WcjE, WciG creates ficolin-2 targets only on serotype 35B. Ficolin-2 binds other *wciG*-encoding serotypes (e.g., serotype 35C), but they frequently encode *wcjE* as well. While ficolin-2 targets are acetyl groups, not all acetyl groups are ficolin-2 targets, and the context of the capsule structure is critical in creating ficolin-2 targets. This point was clearly demonstrated with WcjE as well [8]; ficolin-2 does not bind to serotype 9V even though WcjE acetylates N-acetyl mannose in that structure.

In addition to sharing the propensity for IPD among older adults, serotype 35B shares additional epidemiologic characteristics with serotype 11A. First, they have derivative serotypes, 35D and 11E, which were created by inactivating the genes for an acetyl transferase (*wciG* and *wcjE* respectively). In both cases, the inactivation led to the loss of the target for ficolin-2 and loss of complement deposition in vitro. Moreover, the serotype 11E is preferentially found among IPD isolates, not nasopharyngeal isolates [15]. While serotype 35D was only recently discovered, all known isolates were obtained from individuals with invasive disease [3, 16, 17], and genetic inactivations of *wciG* are often unique to each isolate just like *wcjE* inactivations of serotype 11E. Thus, 35B appears to have microevolved into 35D in each host. Molecular mechanisms used for the microevolution are not yet clear, but all serotype 35D isolates have either inactivating point mutations or small internal deletions resulting in premature termination [3, 16, 17]. Nevertheless, these additional epidemiologic observations support the idea that the derivative serotypes represent mutants that arose to escape ficolin-2 in each individual host.

Although serotype 31 IPD showed an age distribution similar to that of serotypes 11A and 35B, no *wcjE*-deficient derivative of serotype 31 has been described. This absence is likely due to relative rarity of serotype 31 IPD and/or no searches for such a serotype. We do show herein that this hypothetical serotype (31X1) is biologically possible. Next, we could not demonstrate ficolin-2 mediated complement deposition in vitro. For technical reasons, diluted sera were used as complement sources in our assays. As serotype 31 is relatively weakly bound by ficolin-2 in comparison to serotypes 11A or 35B, it may be that ficolin-2 on the serotype 31 bacterial surface was insufficiently dense to trigger complement activation in vitro. However, ficolin-2 may be able to opsonize serotype 31 in vivo (i.e., in the presence of undiluted blood). Alternatively, it is not out of the question that ficolin-2 may indeed fail to activate complement upon serotype 31 and that the observed distribution is coincidental or due to another unknown aging-related factor.

Invasiveness of pneumococci can be epidemiologically determined for a population by measuring carriage and IPD rates in the population. Weinberger et al. used this approach to estimate invasiveness of each pneumococcal serotype in adults older than 40 years of age and children (<5 years) [27]. The study identified serotypes 3, 6A/C, 8 and 11A to be more invasive among adults older than 40 [27]. Also their data suggest that serotype 35B may be more invasive among adults older than 40 (Web Fig 2 in [27]). Given our data, it is possible that children have ficolin-2 mediated immunity to serotypes 11A and 35B but the immunity wanes among adults older than 40.

Taken together, our findings suggest that there may be a breakdown of ficolin-2-mediated immunity among older adults. While we did not observe reduced ficolin-2 levels in older adults [28] and have no molecular basis for our findings at the moment, lectin pathway activation is more complex than activation of the classical pathway. For the classical pathway, IgM or IgG molecules need to attract one C1 complex for activation since the C1 complex has both proteases (C1r and C1s) necessary for the activation. Lectin pathway also needs two different MBL/ficolin associated serine proteases (MASP-1 and MASP-2) to be brought together for activation. Ficolin-3 (H-ficolin), which is very similar to ficolin-2, can complex with only one MASP in its physiological state [24]. Consequently, MASP-1 and MASP-2 must be brought together by two different ficolin/MASP complexes. Further complicating matters, each MASP’s gene has alternative splice variants (MASP-3 and MAp44 for MASP-1 and MAp19 for MASP-2), and MASP-1 and MASP-2 exist in unequal proportions: MASP-2 is much less prevalent than MASP-1 in serum [29]. Thus, it is possible that age related changes in expression may cause some of these components to be limiting in older adults. Broadly, the impact of aging on complement function has been inadequately studied. More complete knowledge is essential to understanding infectious disease progression in an aging population. As there are good epidemiologic data on pneumococcal infections by different serotypes types among different age groups, the myriad serotypes of pneumococci may prove ideal for studying the impact of aging on complement function, and pneumococcal epidemiology may help us understand the roles the lectin pathway plays during infections.

## Supporting information

S1 Fig Ficolin-2 binds to multiple serotype 35B isolates. Indicated strains were assayed for ficolin-2 binding as described in *Methods*. Green bars, serotype 35B isolates; black bar, serotype 11A (positive control); red bar, serotype 11E (negative control); blue bar, serotype 35D; white bar, TIGR4 (negative control). For controls, serotype is indicated in parentheses.
(PDF)

S1 Table Adjusted number of cases of invasive pneumococcal diseases in the United Kingdom from 2000–2006, by serotype and patient age^1^. ^1^Data were obtained from Miller et al. (Lancet Infect Dis 2011: 11,760–768). The manuscript reported “adjusted” case numbers, which were adjusted for missing data or changes in survey population and underlying changes in disease ascertainment.
^2^*P* values were obtained with Fisher’s exact test using “Other Serotypes” as the reference.
(DOCX)
