# Supplementary figures and images for: Ficolin-2 binds to serotype 35B pneumococcus as it does to serotypes 11A and 31, and these serotypes cause more infections in older adults than in children
Source: PLoS One. 2018 Dec 26;13(12):e0209657. doi: 10.1371/journal.pone.0209657 (PMC6306229; doi:10.1371/journal.pone.0209657)

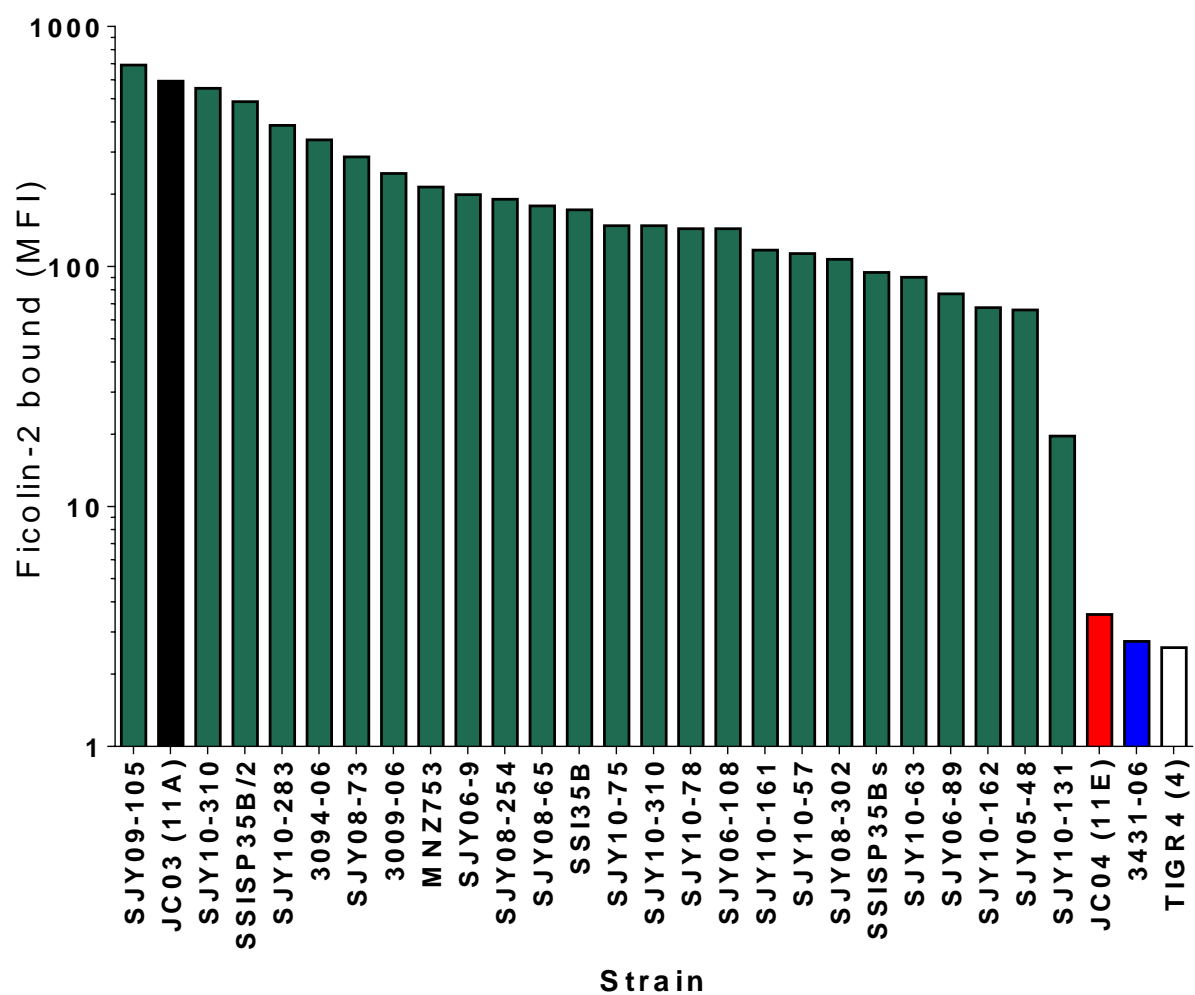

Supplement: S1 Fig — Indicated strains were assayed for ficolin-2 binding as described in Methods. Green bars, serotype 35B isolates; black bar, serotype 11A (positive control); red bar, serotype 11E (negative control); blue bar, serotype 35D; white bar, TIGR4 (negative control). For controls, serotype is indicated in parentheses. (PDF) [file pone.0209657.s001.pdf]
