# Supplementary material for: Ficolin-2 binds to serotype 35B pneumococcus as it does to serotypes 11A and 31, and these serotypes cause more infections in older adults than in children
Source: PLoS One. 2018 Dec 26;13(12):e0209657. doi: 10.1371/journal.pone.0209657 (PMC6306229; doi:10.1371/journal.pone.0209657)
Supplement: S1 Table — 1Data were obtained from Miller et al. (Lancet Infect Dis 2011: 11,760–768). The manuscript reported “adjusted” case numbers, which were adjusted for missing data or changes in survey population and underlying changes in disease ascertainment. 2P values were obtained with Fisher’s exact test using “Other Serotypes” as the reference. (DOCX) [file pone.0209657.s002.docx]

| Serotype | <5 years old | >65 years old | *P* value^2^ |
| --- | --- | --- | --- |
| 11A | 4 | 49 | 0.0013 |
| 31 | 0 | 15 | 0.0165 |
| 35B | 1 | 6 | 0.6852 |
| All other serotypes | 1065 | 3055 | --- |
| Total | 1070 | 3125 | --- |
